# Determinants of Seasonal Elephant Habitat Use in Small and Enclosed Conservation Area: Mwea National Reserve, Kenya

**DOI:** 10.1002/ece3.73538

**Published:** 2026-04-29

**Authors:** Josephat K. Wambua, Yussuf A. Wato, Siro A. Abdallah, John K. Mworia, Catherine Lukhoba, Nathaniel N. Gichuki

**Affiliations:** ^1^ Department of Biology‐Kenya University of Nairobi Nairobi Kenya; ^2^ WWF‐Kenya Nairobi Kenya; ^3^ Meru University of Science and Technology Meru Kenya

**Keywords:** African elephants, environmental variables, habitat use, invasive species, MaxEnt, seasonality

## Abstract

This study explored the drivers of seasonal African savanna elephants ( *Loxodonta africana* ) habitat use in a small, enclosed conservation area with limited space and no migration corridors. It examined how elephant habitat‐use dynamics may influence management strategies, human–wildlife conflict mitigation, and the long‐term sustainability of elephant populations in small and isolated protected areas. The study was conducted in Mwea National Reserve, a fenced protected area in Kenya. Elephant spatial distribution was assessed using indirect signs of presence. We conducted 96 straight‐line dung transects (250 m each) and 350 m reconnaissance walks across the reserve to document dung occurrence and density. Seasonal elephant distribution was modeled using Maximum Entropy (MaxEnt) using five environmental predictors. We used the jackknife method to evaluate the relative influence of each predictor on seasonal habitat use. We hypothesized that (i) water and forage availability would primarily determine dry‐season habitat use, and (ii) landscape variables would be more influential in the wet season, when water and forage are less limiting. MaxEnt models performed moderately well, with Area Under the Curve (AUC) values of 0.714 (dry) and 0.664 (wet). In the dry season, the most influential predictors were invasive plant presence (28.9%), proximity to permanent rivers (26.9%), and elevation (26.5%). In the wet season, distance to the reserve boundary was the strongest predictor (38.9%), while NDVI showed stable influence across seasons (16.2% dry, 20.1% wet). Small and isolated conservation areas holding elephants among other large herbivores pose many ecological and management challenges. In Mwea National Reserve, this study found a rapidly growing elephant population, high density, and seasonal shifts in habitat use, primarily driven by water and forage availability. Land elevation, reserve boundaries, and other infrastructure had limited influence on elephant distribution and food access. However, the distribution of invasive plant species overlapped with key elephant foraging sites, thereby restricting access to usable plants in non‐invaded areas. Controlling invasive plants can improve forage access and relax browsing pressure on natural vegetation. Maintaining fence integrity and sustained cooperation with the surrounding community is crucial to limit foraging range expansion as well as monitor and reduce human–elephant conflict. The findings of this study provide usable information and spatially explicit guidance for managing savanna elephant populations in small and isolated conservation areas.

## Graphical abstract

This study investigated the drivers of seasonal habitat use by African savanna elephants ( *Loxodonta africana* ) in the fenced Mwea National Reserve, using dung‐based surveys and MaxEnt models. Results showed that water and forage availability primarily shaped dry‐season distribution, while boundary effects were more influential in the wet season, with moderate model performance (AUC = 0.714 dry; 0.664 wet). It further found that high elephant density and seasonal habitat shifts, compounded by invasive plant encroachment on key foraging areas, underscore the need for targeted invasive species control, sustained fence maintenance, and community collaboration to support long‐term ecological sustainability and reduce human–wildlife conflict in small, isolated reserves.

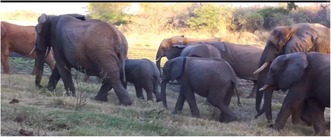

## 1 Introduction

Globally, large herbivores are experiencing significant population declines and range contractions, leading to enhanced risk of extinctions (Ripple et al. 2015). Key drivers of these declines include hunting, land‐use change, human encroachment, and expansion of agricultural land (Ceballos et al. 2017; Ripple et al. 2015). Commercial hunting and illegal trade in products derived from large herbivores, particularly ivory and rhino horn, pose the most severe threats to the survival of the majority of wide‐ranging mega herbivores (Chase et al. 2016; Wasser et al. 2015; Wittemyer et al. 2014).

The Africa savanna elephant ( *Loxodonta africana* ) has undergone drastic population declines due commercial hunting, habitat loss and conflicts with communities (Chase et al. 2016; de Sales et al. 2020; Gross and Heinsohn 2023). Due to increased threat levels, African savanna elephant has been classified as an endangered species (appendix II) by the Convention on International Trade in Endangered Species of wild flora and fauna (CITES). This status demands that international trade in life animals and their products be regulated. Additionally, elephant range states in Africa have implemented a variety of conservation strategies aimed at reversing the declining population trends (Botey et al. 2022; Correa et al. 2024). These strategies include enhanced security measures, deliberate habitat management, and the construction of barrier fences to minimize conflict with humans (Botey et al. 2022; Correa et al. 2024; Huang et al. 2024). Despite adopting the above strategies to reduce mortality, savanna elephant populations continue to decline in many African range states (Chase et al. 2016; Edwards 2024).

One important but often underrated aspect of conservation is the seasonal spatial ecology of savanna elephants—that is, how they use space and select habitats across seasons. Information on habitat use and the factors influencing it is critical for effective management of large herbivores in  a dynamic landscape (Bastille‐Rousseau et al. 2019; Gaston et al. 2000). Habitat use data are additionally essential for conservation planning (Lakshminarayanan et al. 2016), predicting potential human‐wildlife conflict zones (Ashiagbor and Danquah 2017; Mlambo et al. 2024), and supporting herbivore nutritional requirements as well as facilitating behavioral adaptation for overall fitness and reproductive success through acquisition of fundamental resources and threat evasion (Alipertil et al. 2022; Matthiopoulos et al. 2020).

In this context, understanding habitat use and the factors influencing the underlying dynamics is therefore critical (Lakshminarayanan et al. 2016; Mariotti et al. 2020; Mlambo et al. 2024). For example, during the dry season, availability of water (Chui et al. 2024; Mlambo et al. 2024; Wato et al. 2018) and vegetation productivity (Anderson et al. 2016; Clegg and O'Connor 2025; Muposhi et al. 2016) have been shown to influence the distribution of elephants. However, the combined and interactive effects of water access, land elevation and topography as well as habitat‐fragmenting linear infrastructure, such as roads, on habitat use by savanna elephants in small and enclosed conservation areas remain unexplored. Similarly, the influence of resource quantity and quality as well as invasive plant species on seasonal habitat use remains poorly understood. As keystone species, elephants exert cascading ecological impacts on their habitat and it's therefore imperative to understand the drivers of seasonal space use for effective habitat conservation (Ashiagbor and Danquah 2017; Chui et al. 2024; Muposhi et al. 2016).

Several direct and indirect methods have been used to evaluate habitat selection and use by large herbivores (Duparc et al. 2019; Dupke et al. 2021). The direct methods are mainly based on field observations, while the indirect methods rely on detection and counting of signs of animal presence, such as pellets, tracks, bed‐sites, and Global Positioning System (GPS) collars as an index of habitat use by the whole population (Chui et al. 2024; Fragoso et al. 2016). Pellet‐group count methods have been widely used in studies of habitat use by red deer ( *Cervus elaphus* ) (Alves et al. 2014) and moose ( *Alces alces* ) (Månsson et al. 2011), while dung piles have been widely used to evaluate habitat selection by Asian elephants ( *Elephas maximus* ) (Lakshminarayanan et al. 2016). GPS‐enabled collars have also been extensively used in Africa to study local movement or foraging ranges of large herbivores, such as elephants and rhinoceros (Mukomberanwa et al. 2025; Muposhi et al. 2016). However, collars are expensive, and their cost can limit sample sizes and influence the foraging or hunting behavior of the collared individuals (Van De Bunte et al. 2021; İmamoğlu and Taş 2022). The use of signs such as dung and footprints offers a reliable non‐invasive method because signs are generated by the targeted species (Fragoso et al. 2016; Peralta et al. 2023). To convert these signs into spatially explicit patterns of habitat use, Geographic Information Systems (GIS) software and associated analytical tools are often used (Elith et al. 2006).

In this study, we used the MaxEnt algorithm to model the spatial distribution of elephants during the dry and wet seasons using elephant dung deposits at different decay statuses to explore the environmental and landscape variables that influenced seasonal habitat use within Mwea National Reserve. The key predictor variables used were: reserve boundaries, land elevation, invasive species, Normalized Difference Vegetation Index (NDVI), and distance to the water source. The choice of MaxEnt was guided by its efficiency and robustness in predicting elephant distribution from presence‐only data and high predictive power compared to other methods (Elith et al. 2006, 2011). Furthermore, MaxEnt and GIS tools have been used elsewhere in Africa to gain insights on ecological, landscape, and anthropogenic factors influencing habitat use by elephants (Ashiagbor and Danquah 2017; Mlambo et al. 2024).

With the tropical dry savanna climate prevailing in Mwea National Reserve, we hypothesized that dry‐season elephant habitat use would be mainly determined by the availability of water and forage resources while wet‐season habitat use would be adversely influenced by landscape attributes,such as land elevation and slope as well as the density of invasive plants, linear infrastructure, and reserve boundaries. Understanding these drivers and their influence on habitat use by elephants is fundamental for producing evidence‐based conservation strategies that enhance habitat integrity, promote the viability of the target elephant population, and mitigate human–wildlife conflict, thus contributing to the long‐term existence of isolated elephant populations in Africa.

## 2 Methodology

### 2.1 Study Area

**FIGURE 1:**
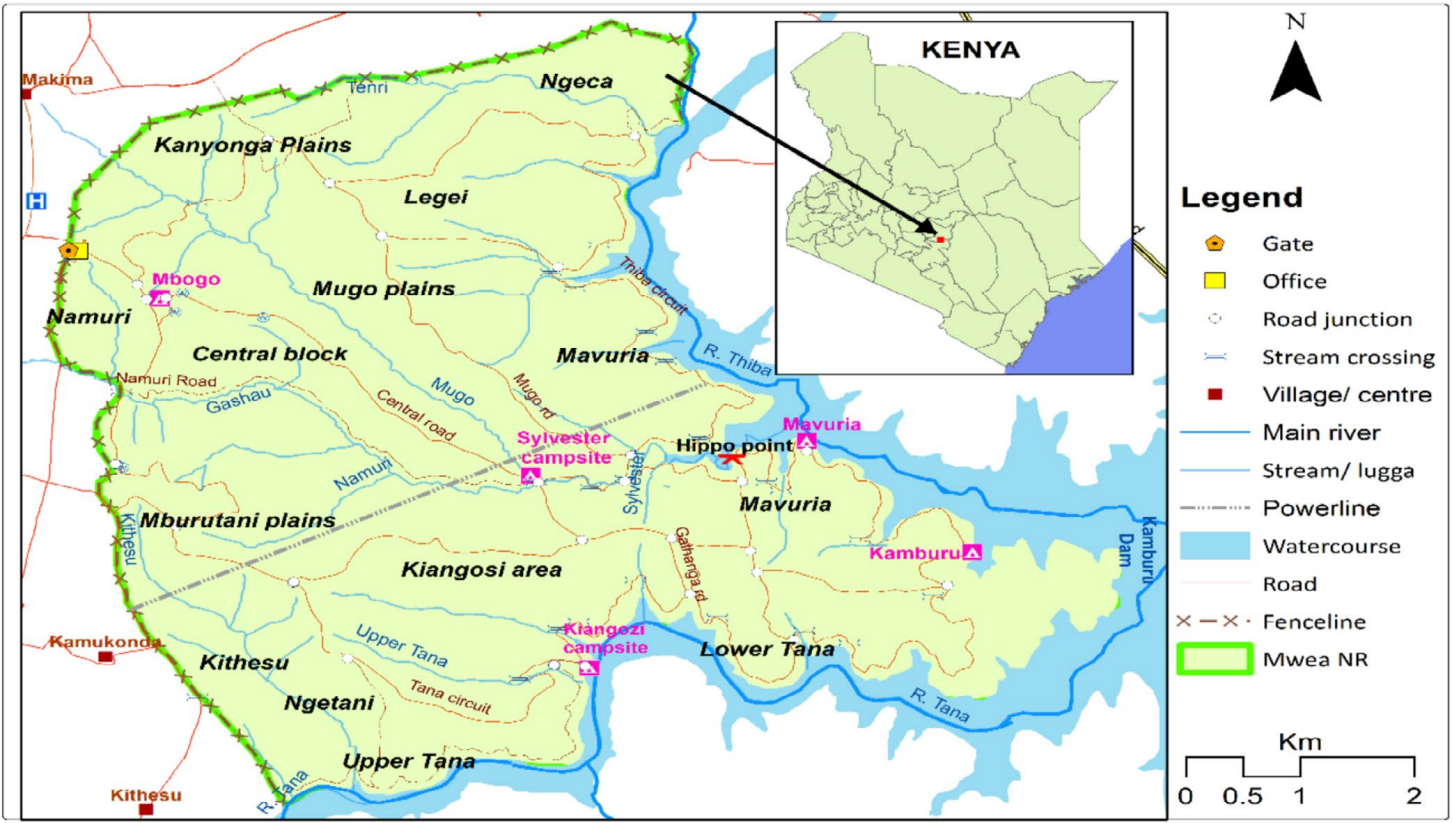
Map of Mwea National Reserve showing its geographical location in Kenya.

This study was conducted in Mwea National Reserve, a 44 km^2^ conservation area located at latitudes 0° 45′ and 0° 52′ South and longitudes 37° 35′ and 37° 40′ East, and lies at an altitude of 950–1150 m above sea level in Embu County‐Kenya (Figure 1). One side of the reserve boundary is marked by an artificial fence line while the other boundaries are formed by two permanent rivers (Thiba and Tana), whose confluence is at Kaburu dam. The land is predominantly covered by sandy soils and gently slopes towards the two rivers and the dam. The geological setup comprises granitic and igneous bedrock. The climate is semi‐arid with annual rainfall ranging from 510 to 760 mm. Annual rainfall distribution is bimodal, with peaks expected in April and November, but can be poorly distributed. Mean minimum and maximum temperatures are 14°C and 30°C, respectively. The vegetation of the reserve falls under eco‐zone five (Pratt et al. 1966), and is characterized by minor changes over a short distance in response to changes in local topography and soil types. There are five different plant communities in Mwea National Reserve, comprising *Senegalia mellifera* woodland, wooded shrub lands, C*ombretum apiculatum* wooded grassland, riverine/riparian woodland and undifferentiated vegetation in degraded areas. The woodlands are dominated by *Senegalia mellifera*, shrub‐lands by *Barleria acanthoides*, wooded grasslands by *Combretum apiculatum*, riverine woodland by *Acacia elata* while the degraded area has patches of *Euphorbia robecchii* . Invasive plant species occur in small patches but their distribution tends to be continuous where ground water is close to the soil surface, such riparian zones.

### 2.2 Data Acquisition and Pre‐Processing

#### 2.2.1 Seasonal Elephant Location Data

Seasonal elephant habitat use was assessed indirectly through dung count surveys in 96 straight‐line transects, each measuring 250 m in length, systematically distributed across the entire Mwea National Reserve. To enhance spatial coverage, we also conducted reconnaissance (recce) walks, measuring approximately 350 m between successive transects, following paths of least resistance to search for additional dung signs. The recces served as supplementary indicators of elephant presence and habitat use. Transect locations were determined using a systematic grid‐based approach by dividing the study area into 600 m × 600 m grid cells using ArcGIS (ESRI, Redlands, CA). The resulting grids were overlaid onto a topographic map of the reserve. Each grid cell was labeled for reference. The center point of each grid cell was identified using ArcGIS spatial tools and recorded as the starting point of the corresponding 250 m transect.

**FIGURE 2:**
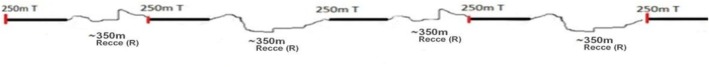
Horizontal profile of 250 m transects interspersed with 350 m recces used for elephant habitat‐use determination in Mwea National Reserve.

Using standardized field data sheets and hand‐held global positioning system (GPS) receivers, we recorded elephant dung that could be seen on either side of each 250 m transect and associated recce walk (Figure 2). Transects were oriented in an east–west or west–east direction. To minimize sampling bias, transect walks were done perpendicularly to elephant trails, rivers, and roads. For each elephant dung pile seen on either side of the 250 m transects and the 350 m recce paths, we recorded GPS coordinates and classified the dung into one of five age categories, ranging from Class A (fresh) to Class E (oldest) (Barnes 2001; Hedges 2012). Data on elephant habitat use were collected during both the dry and wet seasons following the survey procedures outlined above.

#### 2.2.2 Pre‐Processing of Elephant Dung Data

GPS waypoints for elephant dung locations were downloaded and merged with corresponding dung class data in ArcGIS to create a unified dataset indicating the precise location and age classification of each dung pile. From the merged dataset, a total of 2540 elephant dung waypoints were extracted for the dry season (July–October) and 1345 waypoints for the wet season (November–January). These waypoints were subsequently used as spatial indicators of elephant habitat use during the respective seasons in Mwea National Reserve.

For the purpose of analyzing seasonal habitat use, waypoints for dung classified as Class A to Class C2 were used, as these categories represent relatively recent defecation events and habitat use. Elephant dung piles falling under category D and E were few and hence were excluded from the analysis as they were considered too old to reliably indicate recent elephant presence.

#### 2.2.3 Environmental Variables

To model elephant habitat suitability, five environmental variables were selected based on their ecological significance, landscape characteristics, and availability of spatial data. These variables were hypothesized to influence seasonal habitat use and the spatial distribution of elephants within the study area. Their importance in shaping elephant habitat selection has been well‐documented in previous studies (Ashiagbor and Danquah 2017; Bastille‐Rousseau et al. 2019; Bohrer et al. 2014; Tsalyuk et al. 2019). The selected variables included Normalized Difference Vegetation Index (NDVI) mean as an indication of primary productivity, distance to main rivers, distance to national reserve boundary, elevation, and invasive species distribution.

##### 2.2.3.1 Distance From the Reserve Boundary

This variable established the Euclidean distance from the reserve boundary as an indication of anthropogenic edge effects and disturbance on the distribution of elephants. Generally, elephants avoid humans and their activities and this has an impact on how they access and use resources within the ecosystem (Buchholtz et al. 2021). Moreover, disruptive human activities and developments can cause wildlife to avoid certain areas (Doherty et al. 2021; Gaynor et al. 2018; Jaeger et al. 2005), influencing spatial use.

##### 2.2.3.2 Elevation (Digital Elevation Model)

Land elevation, derived from a Digital Elevation Model (DEM), was included as a key topographic variable due to its influence on vegetation structure, water availability, and temperature gradients, all of which can affect elephant movement patterns and habitat choice (Bohrer et al. 2014). Lower elevations typically support more productive and water‐rich vegetation, particularly valuable during the dry season, while higher elevations may become accessible and ecologically beneficial during the wet season when ephemeral water sources and forage become available (Loarie et al. 2009). Elevation indirectly reflects terrain accessibility, as elephants tend to avoid steep or rugged slopes, particularly when resources available there are limited (Wall et al. 2006).

##### 2.2.3.3 Invasive Species Distribution and Abundance

Invasive plant species have been demonstrated to reduce forage availability for herbivores by displacing native vegetation, altering plant community composition, and reducing habitat quality (Lahkar et al. 2011; Oduor et al. 2018; Siddiqui et al. 2021). In Mwea National Reserve, the major invasive species were *Parthenium hysterophorus* , *Senna didymobotrya* , and *Senna spectabilis* , all of which are known to aggressively colonize disturbed areas and suppress native plant regeneration. To assess the distribution of these invasive species, data were collected using purposive sampling in 85 randomly located 5 m × 5 m plots. Each plot was surveyed for the presence, abundance, and dominance of the target invasive species and its waypoint documented using a GPS. This information provided a spatial dimension of invasion patterns and their potential impacts on elephant forage availability and habitat use.

##### 2.2.3.4 Seasonal Mean of the Normalized Difference Vegetation Index (NDVI)

Availability and variability of forage in African savannas during the dry season is a critical driver of habitat suitability and elephant space use (Fullman et al. 2017). NDVI is a reliable representation of forage richness for elephants (Muposhi et al. 2016), and is a popular index for estimation of vegetation vigor or greenness (Duffy and Pettorelli 2012), its derived from near‐infrared and red bands of satellite imagery.

(1) $$\text{NDVI}=\left(\mathrm{NIR}-R\right)/\left(\mathrm{NIR}+R\right)$$

In this study, Sentinel 2, freely available satellite imagery at 10 m spatial resolution, was used to extract NDVI seasonal mean data of the study area.

##### 2.2.3.5 Distance From Surface Water Sources

Savanna elephants tend to forage in areas close to water sources, thereby balancing drinking and feeding needs. Water availability is therefore a key determinant of how elephants utilize space, particularly in semi‐arid ecosystems (Howes et al. 2020; Sach et al. 2019). Distance to water sources data were processed by running the Euclidean distance tool in ArcGIS pro.

#### 2.2.4 Processing of Environmental Variables

Environmental variables were converted into raster format using ArcGIS (Esri 2020). Each raster was resampled to a spatial resolution of 500 m to ensure uniformity in scale and extent across the study area. The rasters were clipped to the boundaries of the reserve using the reserve boundary shape‐file. To ensure compatibility with Maximum Entropy (MaxEnt) model, all raster layers were transformed into American Standard Code for Information Interchange (ASCII) format and projected using Universal Transverse Mercator (UTM) zone 37 South coordinate system and resampled at a 30 m spatial resolution to ensure compatibility for spatial modeling. These steps were implemented using ArcGIS Model‐Builder to enhance reproducibility and streamline workflow processes.

## 3 Data Analysis

### 3.1 Modeling Spatial–Temporal Distribution of Elephants

MaxEnt machine learning algorithm was used to model the seasonal distribution of elephants in Mwea National Reserve based on presence‐only records (Elith and Graham 2009) derived from dung pile locations as well as environmental and landscape variables that were hypothesized to influence seasonal space use by elephants in the reserve.

We evaluated the performance of seasonal habitat use model using the Area Under the Curve (AUC) of the Receiver Operating Characteristic (ROC) plot, with AUC values interpreted based on Baldwin (2009) criteria as not better than random, fair‐model, and good‐model, which translates to an AUC range below 0.5, between 0.5 and 0.7, and 0.7–0.9. The MaxEnt modeled seasonal space use data generated a continuous habitat suitability map, with values ranging from 0 (unsuitable) to 1 (highly suitable). These results were further analyzed using GIS to identify spatial patterns and elephant utilization zones across the two seasons within the reserve.

### 3.2 Testing for Multicollinearity

High collinearity among the predictor variables often causes model overfitting (Pradhan 2016) in statistical species distribution modeling. In this study, we tested for collinearity (high levels of interdependence among predictors in a regression model) before their use in MaxEnt elephant distribution modeling using variance inflation factors (VIFs) (Akinwande et al. 2015). The analysis was conducted by employing the Spatial Statistics toolbox in ArcGIS Pro (version 10.1), specifically the “Collinearity Diagnostics” tool available under the Generalized Linear Regression (GLR) framework. VIF values quantify how much the variance of an estimated regression coefficient is increased due to collinearity.

To assess multicollinearity among predictor variables, Variance Inflation Factor (VIF) values were interpreted based on standard thresholds: VIF values greater than 7.5 indicated high collinearity, suggesting the need to consider expunging the associated variables; values between 2 and 7.5 were considered to reflect moderate collinearity, which may be acceptable depending on the context; and values below 2 were regarded as indicative of low collinearity, which is generally desirable in line with established statistical practice.

Table 1 summarizes the VIFs of the predictor variables after collinearity diagnostics. In both dry and wet seasons, all the variables met the required threshold of the model (< 7.5). Five exploratory variables were therefore included in the MaxEnt model for the potential elephant habitat use in the reserve for the two seasons.

**TABLE 1:** Summary results from variables multicollinearity assessment.

Variable	Dry season				Wet season			
	VIF	% Significant	% Negative	% Positive	VIF	% Significant	% Negative	% Positive
Boundary distance	1.68	100	100	0	1.46	100	0	100
Elevation	2.69	100	100	0	1.98	75	75	25
Invasive species	1.37	100	100	0	1.19	100	0	100
NDVI mean	1.02	100	0	100	2.05	100	0	100
Main river distance	2.54	93.75	87.5	12.5	1.12	93.75	50	50

## 4 Results

### 4.1 Seasonal Space Use and Potential Distributions of Elephants

**FIGURE 3:**
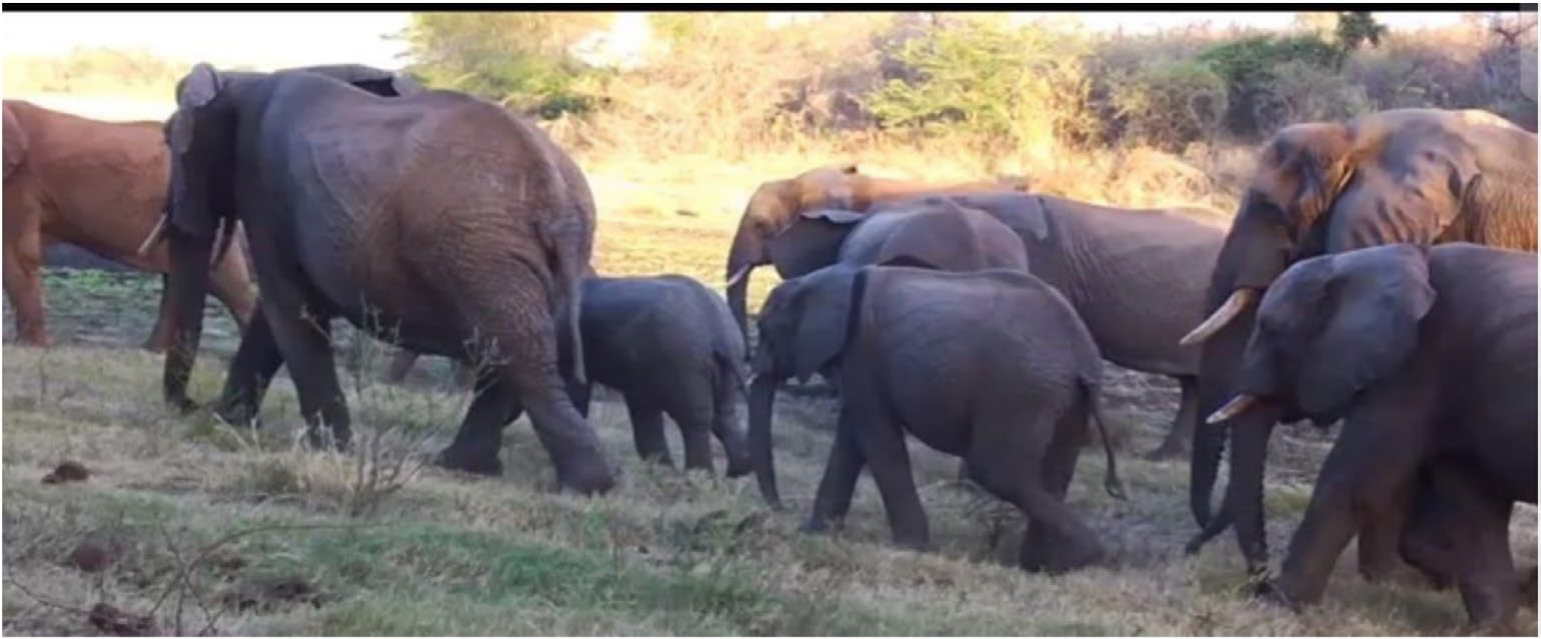
African Savanna Elephants ( *Loxodonta Africana* ) utilizing riparian area in Mwea National reserve.

**FIGURE 4:**
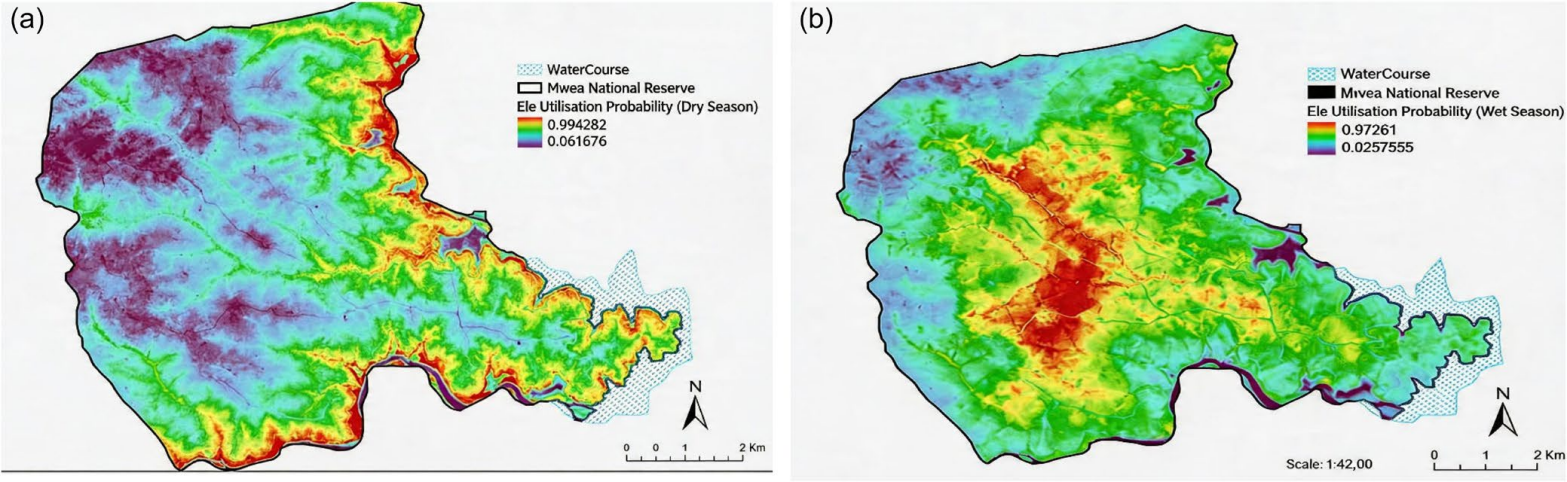
Predicted seasonal spatial distribution of elephants in Mwea National Reserve, Kenya.

During the dry season, elephant utilization zones were concentrated around the two key permanent river systems to the east (Tana River) and south of the reserve (Thiba River) and the riparian area around Kaburu dam (Figure 3). In the wet season, utilization zones were within the bush land found in the central parts of the reserve. The predicted spatial distribution of elephants modeled using MaxEnt for the dry season (a) and wet season (b) is shown in Figure 4.

**FIGURE 5:**
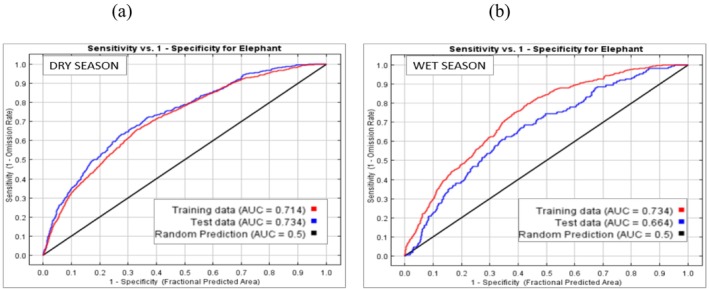
Showing the AUC model evaluation results for dry and wet seasons.

The species distribution models (dry season and wet season) employed in this study registered an AUC between 0.714 and 0.664 respectively (Figure 5), indicating that the model used was fair and capable of predicting the environmental factors that had significant influence on space use by elephants in the reserve.

### 4.2 Environmental Variables Influencing Elephant Distribution

**FIGURE 6:**
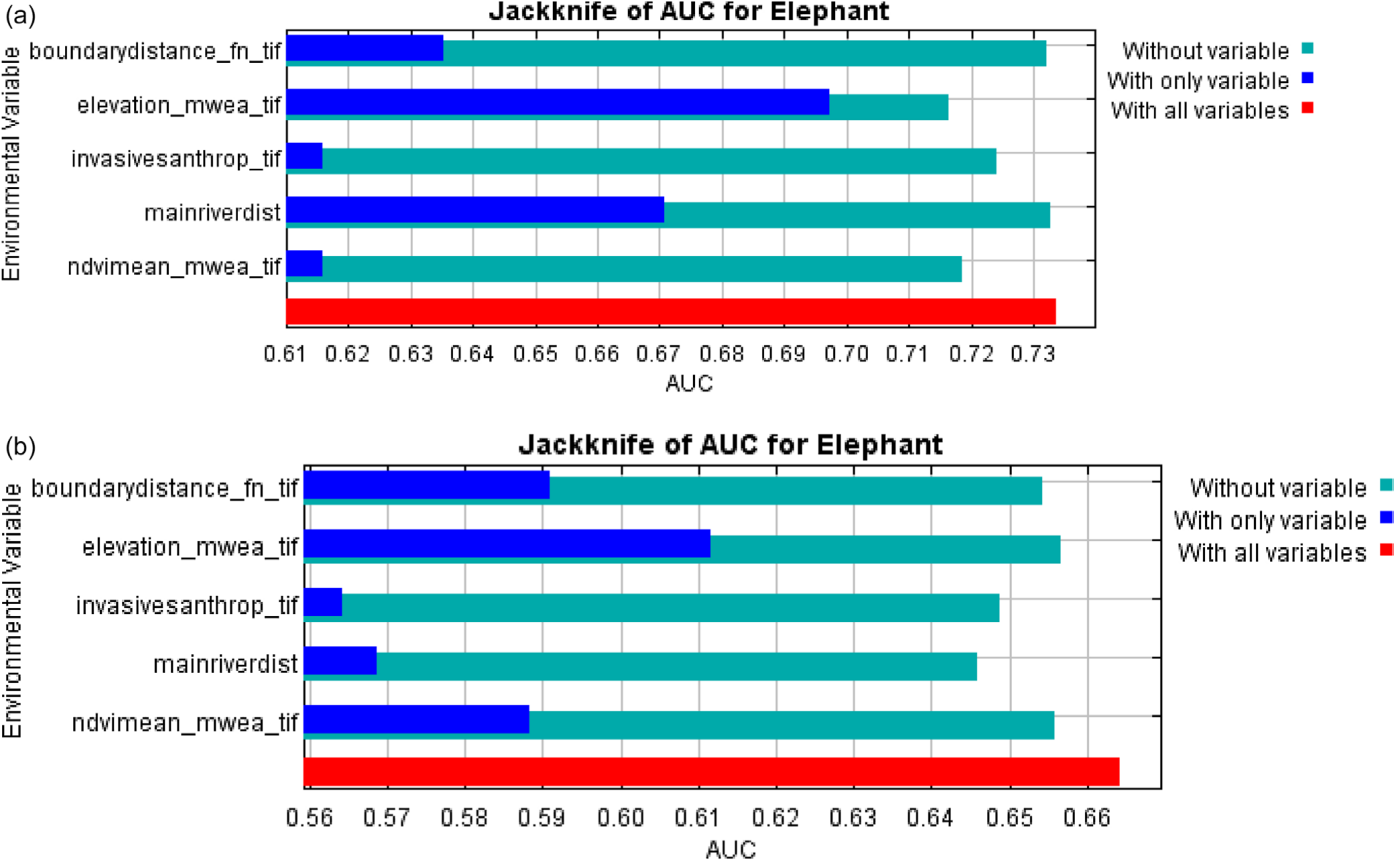
Showing jackknife test results for variable importance. (a) Dry season jackknife variables contribution to elephant distribution in Mwea National Reserve. (b) Wet season jackknife variables contribution to elephant distribution in Mwea National Reserve.

The percentage contribution of the variables influencing spatial distribution of elephants as well as Jackknife tests generally showed consistent patterns, with minor variations attributable to differences in the methods used to estimate variance. For example, in the dry season invasive species contributed 28.9% to the variance in model prediction, followed by distance to permanent water sources at 26.9% and land elevation at 26.5%. In the dry and wet seasons, mean NDVI's values remained relatively stable and their contribution to variation in model prediction was 16.2% and 20.1%, respectively. Surprisingly, distance to the reserve boundary had the highest contribution at 38.9% during the wet season. The contribution to model prediction variation by other environmental variables was moderate except for the mean NDVI, which reflected seasonal food resource changes and consequential changes in habitat use by elephants in the reserve (Table 2 and Figure 6).

**TABLE 2:** Seasonal percentage contribution of predictor variables to elephant space use in Mwea National Reserve.

Season	Boundary distance	Elevation	Invasive species	Main river distance	Mean NDVI
Dry season	1.4	26.5	28.9	26.9	16.2
Wet season	38.9	13.2	16.3	11.6	20.1

## 5 Discussion

This study investigated seasonal habitat use by a population of about 174 elephants resident in a small and enclosed reserve in central Kenya, where rapid population growth has led to overcrowding and frequent conflict with the surrounding farming community. Habitat selection showed marked seasonal variation, primarily influenced by established factors such as water availability (Rech et al. 2025; Sach et al. 2019), forage quality (NDVI) (Gara et al. 2017; Loarie et al. 2009), elevation (Ngene 2010), and landscape features like roads and barriers (Hering et al. 2022; Loarie et al. 2009; Vanak et al. 2010). However, the proliferation of invasive plant species within key resource areas introduces a complex ecological dimension, especially in small, isolated reserves surrounded by community‐influenced landscapes. Because invasive species such as *Parthenium hysterophorus* (Wambua et al. 2025) and *Lantana camara* (Oduor et al. 2025; Stewart et al. 2021, 2025) are known to reduce native forage species availability and degrade habitat quality particularly within riparian and stream habitats that are used as dry season grazing areas (Fynn et al. 2015), their effect in small and isolated conservation areas can have adverse effects on food resources for endangered species such as the African savanna elephants (Wambua et al. 2025).

During the dry season, elephants were concentrated in the wooded riparian zone of two permanent rivers and the margins of Kaburu Dam. However, in the wet season, elephants expanded their range into the central sections of the study area, which was dominated by scattered trees, shrubs, and tall grasses. These results indicate a hierarchical habitat selection structure determined by water access, forage availability, and land topography. Distance to the reserve boundary was equally a strong determinant of space use in the wet season due to the constraining effect of the fence barrier. Seasonal segregation in habitat selection by savanna elephants has been reported widely (Lakshminarayanan et al. 2016; Mukomberanwa et al. 2024; Owen‐Smith and Chafota 2012; Yoganand and Owen‐Smith 2014), its believed to be an adaptation for accessing adequate nutrient‐rich food, especially food that is rich in nitrogen, phosphorus, and sodium throughout the year.

Water availability was the dominant determinant of dry‐season elephant habitat use as shown by the strong performance of river distance in the MaxEnt model. This emphasizes water access as a critical factor limiting dry season habitat use. This finding is consistent with the high daily water requirements by elephants for maintaining physiological function (Davis et al. 2022; de Knegt et al. 2011; Wato et al. 2018) and is associated with high daily water requirements by elephants for maintaining physiological function. As surface water became spatially restricted following the drying of ephemeral streams, water pans and waterholes, elephants concentrated near permanent water sources and riparian habitats which typically contain browse plants and sedges that remain green during dry periods, providing reliable foraging resources, cover, and shade (Anderson et al. 2016; Mlambo et al. 2024). Studies by Sach et al. (2019), emphasize the importance of access to drinking water and food moisture through their influence on spatial distribution of elephants during the dry season. For example, lactating females with young calves are known to be highly dependent on water; as such, their ranging patterns are strongly influenced by water availability and constraints associated with parental care of their young (Chui et al. 2024; Ndaimani et al. 2017).

Normalized Difference Vegetation Index (NDVI) contributed substantially to habitat use during the dry season. As NDVI indicates potential food for elephants and other browsing herbivores, high NDVI values in the riparian zones of water sources could reflect vegetation productivity and availability of nutrient rich forage (Sach et al. 2019; Tsalyuk et al. 2019). However, NDVI has been shown to be a coarse measure of food abundance and quality due to uncertainty of food species present relative to all other plant species in the same area (Gautam et al. 2019). Moreover, green vegetation as indicated by high NDVI values, may provide basic browse required to meet dietary needs when preferred forage is generally dry and sparsely distributed in the landscape. In the wet season, increased primary productivity within the entire study area and water availability from ephemeral water‐bodies enabled elephants to exploit a broader landscape. This indicates the importance of food resources in determining the distribution of large herbivores (Borowik et al. 2013; Mariotti et al. 2020). Our results are consistent with Chankhao et al. (2022), and Devi et al. (2022) who found habitat use by large herbivores to be influenced by the availability of principal forage plants.

During this study, it was found that the occurrence and distribution of invasive plant species influenced elephant space use, especially during the dry season. None of the invasive plant species present in the reserve formed an important part of the elephant's diet. However, elephant foraging ranges overlapped with the distribution of the invasive plant species in the reserve. Despite the findings, the influence of the invasive plant can be interpreted in the context of their spatial overlap with the non‐invasive plant species used as food by elephants, especially in the riparian zones of water bodies. Thus, elephants moved to the riparian zones where suitable forage was available, and incidentally, invasive plant species were also abundant in the same areas due to the year‐round presence of moist soil and recurrent disturbances resulting from regular flooding of the riverine areas.

Some invaded areas were utilized by elephants because they held critical dry‐season resources, such as surface water and tree shade cover. Invasive plant species have been shown to reduce the diversity of local palatable species thus limiting the foraging opportunities for most native herbivores (Siddiqui et al. 2021; Stewart et al. 2021). Furthermore, conservation areas surrounded by human‐modified landscapes such as Mwea National Reserve have a high risk of invasive plants Proliferation due to land uses that may provide propagules of invading species (Foxcroft et al. 2017; Spear et al. 2013), with drainage systems of rivers providing pathways for long‐distance dispersal of non‐native species (Foxcroft et al. 2010, 2017). With increasing human footprint within the proximity of many protected areas and changing land‐use practices, the effects of invasive species have potential of displacing palatable forage leading to herbivores nutritional stress.

Elevation, on the other hand, played a secondary role in moderating the spatial distribution of elephants in Mwea National Reserve due to topographic effects on soil moisture retention and forage availability during dry periods (Ashiagbor and Danquah 2017; Makati et al. 2025). Furthermore, the co‐occurrence of lowland elevation and reliable food resources within riparian vegetation may have influenced habitat selection by elephants. In the wet season, low‐lying areas became poorly drained or flooded and hence less suitable for elephant use (Bucciarelli et al. 2024; Chibeya et al. 2021). The seasonal differences in the contribution of land elevation to elephant habitat selection reflect changes in accessibility and primary productivity of the ecosystem.

Reserve boundaries are usually cleared of woody vegetation to ease movement during monitoring and control of break outs by the wild animals and break‐in by humans. Boundaries had a minor effect on habitat use during the dry season because elephants foraged away from the boundaries. This behavior may indicate avoidance of conflict with humans outside the reserve and lack of suitable forage. Elephant concentration in the riparian zones implies that riparian zones offered safety and had the required food resources. Regular use of the riparian zones may also indicate that suitable food resources are depleted in other parts of the reserve and may serve as a safe environment for juveniles (Buchholtz et al. 2021; Dagtekin et al. 2024; Davies et al. 2021). The two rivers and the large Kaburu Dam form natural barriers, and fencing of the northern part of the reserve restricts seasonal movement or dispersal of elephants outside the reserve. This can result in intensified habitat use in key areas within the reserve and may explain local concentration near the riparian zones during the dry season (Fynn et al. 2015; Yoganand and Owen‐Smith 2014). However, competition for food resources by elephants in the reserve eased during the wet season as indicated by the sparse distribution away from riverine areas.

## 6 Conclusion

This study demonstrated that seasonal environmental variability is the primary driver of habitat use by African savanna elephants. With the growing population of this keystone species in Mwea National Reserve, overcrowding could ultimately trigger density‐dependent competition for food resources, which can lead to habitat changes that may not adequately support the survival and reproductive success of the resident elephant population. Habitat selection was still strongly influenced by access to permanent water sources and areas of relatively high vegetation productivity (NDVI). Land elevation and invasive species influenced space use indirectly by limiting access to food resources in some parts of the reserve. There was no preference for utilizing areas invaded by invasive plants, but the latter were spatially linked to water sources and high productive riparian areas. In the wet season, rainfall increased primary productivity in the reserve, thereby reducing dependence on localized food resources. This enabled elephants to expand their foraging range into higher elevations from the low lands near water sources. However, natural and artificial barriers constrained wider dispersal outside the reserve. The stronger boundary effect in the wet season, compared to the dry season, reflected the restrictive role of physical barriers in shaping elephant movement when ecological constraints are relaxed.

Overall, small and isolated conservation areas offer limited habitat options for large herbivores. The observed seasonal shifts in habitat use by Savanna elephants in Mwea National Reserve reflect the central role of forage availability and surface water distribution in a landscape where natural dispersal is restricted. Invasive plant species in the reserve are not widespread but occur in sites used by elephants for foraging. However, the invasive plants have the potential to adversely alter habitat structure, thereby limiting access to feeding sites and concentrating elephants in the few remaining areas with suitable forage. Elephant concentration can lead to localized over‐browsing and further degradation of native vegetation. These changes can intensify pressure on natural vegetation and lead to an increasing risk of human–elephant conflict. Management actions aimed at safeguarding access to key dry‐season water and forage resources, maintaining vegetation heterogeneity, and implementing targeted habitat interventions, such as the eradication of invasive plant species within the reserve, should be prioritized. In order to minimize natural vegetation damage and facilitate its regeneration; and maintain the optimal carrying capacity of elephants in the reserve, it is necessary to regularly translocate some elephants to other conservation areas. These measures are essential to ensure sustainability wildlife living in small and isolated downstream conservation area, such as Mwea National Reserve, where forage resources for elephants may be gradually diminishing and movement options remain limited. Further, a multi‐sectoral approach, including surrounding community engagement, is necessary to address the threats posed by invasive species.

## Author Contributions

**Josephat K. Wambua:** conceptualization (lead), data curation (equal), formal analysis (equal), investigation (equal), project administration (equal), writing – original draft (equal). **Yussuf A. Wato:** formal analysis (equal), validation (equal), visualization (equal). **Siro A. Abdallah:** formal analysis (equal), software (equal), visualization (equal). **John K. Mworia:** conceptualization (equal), methodology (equal), supervision (equal), writing – review and editing (equal). **Catherine Lukhoba:** supervision (equal), writing – review and editing (equal). **Nathaniel N. Gichuki:** supervision (equal), validation (equal), writing – review and editing (equal).

## Funding

The authors have nothing to report.

## Ethics Statement

This study was approved by the National Commission for Science, Technology and Innovation (NACOSTI) license number 629485.

## Conflicts of Interest

The authors declare no conflicts of interest.

## Data Availability

The data that support the findings of this study are publicly available from URL: https://doi.10.1002/ece3.73538.
